# Análise antropométrica dos implantes nacionais e importados de artroplastia total de joelho na população brasileira

**DOI:** 10.1055/s-0045-1812026

**Published:** 2025-11-21

**Authors:** Márcio de Castro Ferreira, Carlos Eduardo da Siveira Franciozi, Luiz Felipe Morlin Ambra, Enzo Salviato Mameri, Marcelo Seiji Kubota, Marcus Vinícius Malheiros Luzo

**Affiliations:** 1 Grupo de Cirurgia do Joelho, Departamento de Ortopedia e Traumatologia, Escola Paulista de Medicina, Universidade Federal de São Paulo, São Paulo, SP, Brasil.; 2 HCor, São Paulo, SP, Brasil.

**Keywords:** articulação do joelho, artroplastia de substituição, doenças articulares, arthroplasty, replacement, joint disease, knee joint

## Abstract

**Objetivo:**

Avaliar a conformidade anatômica de 25 implantes de artroplastia total de joelho (ATJ) à morfologia de joelhos na população brasileira feminina e masculina.

**Métodos:**

Foram analisados 500 exames de ressonância de joelhos de 250 mulheres e 250 homens. As medidas anteroposterior (AP) e mediolateral (ML) dos fêmures, tíbias, e implantes foram tomadas a fim de obter correspondência morfológica.

**Resultados:**

As divergências médias das medidas AP e ML entre as morfologias articulares e os implantes analisados foram as seguintes: fêmures femininos 4,48 mm; fêmures masculinos 4,89 mm; tíbia feminina 3,63 mm; e tíbia masculina 6,11 mm. Os implantes com melhor adaptação AP versus ML foram: Medacta Sphere para o fêmur feminino; Stryker Triathlon para fêmur o masculino; Smith & Nephew Legion para tíbia feminina; e Zimmer Persona para a tíbia masculina. Na comparação da melhor relação de implantes femorais e tibiais para o sexo feminino, observou-se que o United Orthopedic U2 apresentou a melhor pontuação estatística, seguido dos implantes Aesculap Columbus e Smith & Nephew Legion. Para os homens, o melhor escore foi encontrado no Zimmer Persona, Microport Advance e Smith & Nephew Legion. A pior relação feminina foi com o Peter Brehm BPK-S, e a masculina, com o Orthovasive Indus.

**Conclusão:**

Os implantes estudados foram satisfatórios para a cobertura óssea articular dos joelhos de brasileiros de ambos os sexos submetidos à ATJ. No entanto, também foram encontrados resultados com diferenças maiores do que 10 mm na maioria dos implantes. Isso ressalta a necessidade de atenção dos cirurgiões para realizar um bom planejamento para a escolha dos implantes. Demonstrou-se que os implantes importados eram mais customizáveis do que os nacionais na população brasileira.

## Abstract (en)

**Objective:**

To evaluate the anatomical conformity of 25 total knee arthroplasty (TKA) implants to the knee morphology of the Brazilian female and male population.

**Methods:**

We analyzed 500 magnetic resonance imaging (MRI) scans of knees from 250 women and 250 men. We collected data on anteroposterior (AP) and mediolateral (ML) measurements of femurs, tibias, and implants to assess their morphological correspondence.

**Results:**

The mean AP versus ML divergence between joint morphologies and the implants was as follows: 4.48 mm for female femurs; 4.89 mm for male femurs; 3.63 mm for female tibias; and 6.11 mm for male tibias. The implants with the best AP versus ML adaptation were: Medacta Sphere for female femurs; Stryker Triathlon for male femurs; Smith & Nephew Legion for female tibias; and Zimmer Persona for male tibias. When comparing the best femoral and tibial implant ratios for female subjects, the United Orthopedic U2 presented the best statistical score, followed by the Aesculap Columbus and Smith &Nephew Legion implants. For male patients, the implants with the highest scores were the Zimmer Persona, the Microport Advance, and the Smith & Nephew Legion. The worst ratios were found in the Peter Brehm BPK-S for female individuals and the Orthovasive Indus for male subjects.

**Conclusion:**

The implants studied presented satisfactory results for bone coverage of the knees of Brazilian subjects of both genders undergoing TKA. However, we also found differences higher than 10 mm in most implants. This finding highlights that surgeons must carefully plan the TKA during implant selection. Imported implants proved to be more customizable than Brazilian ones in this population.

## Introdução

A artroplastia total do joelho (ATJ) é o tratamento de escolha em pacientes com osteoartrite avançada. 1 2 3 4 5 6 O dimensionamento dos implantes utilizados na ATJ é desenvolvido baseados em estudos de morfológicas ósseas de grupos populacionais baseados principalmente nas razões anatômicas articular entre as distâncias anteroposterior (AP) e mediolateral (ML) dos joelhos. 7 8 9 10

Estudos em diversas populações mundiais como caucasianos, estado-unidense, asiáticos e europeus têm apresentados resultados variáveis para os aspectos morfológicos dos joelhos demonstrando que os implantes disponíveis podem divergir às relações anatômicas de diversas populações étnicas. 7 11 12 13 14

O objetivo deste estudo é avaliar a relação de conformidade anatômica de implantes de ATJ em relação a morfologia de joelhos na população brasileira, a fim de identificar quais produtos se adequam melhor aos joelhos desta população.

## Materiais e Métodos

O estudo foi aprovado pelos dois comitês de ética das instituições envolvidas sob os números (CAAE) 71751923.0.0000.5505 e 79484424.0.0000.0060. Foram selecionados de maneira aleatória e anônimas 500 exames de ressonância magnética (RM) dos joelhos de 250 mulheres e 250 homens.

O critério de inclusão foi a maturidade esquelética de pacientes de ambos os sexos. Os critérios de exclusão foram exames com deformidade morfológica óssea do joelho (osteófitos), dispositivos médicos implantáveis e evidência de fratura articular prévia.

Os pacientes elegíveis foram identificados pelo banco de imagens de RM do Hospital Hcor disponíveis em sua plataforma e software Clinical Collaboration Platform (Carestream Health, Inc.). No caso de pacientes com exames bilaterais disponíveis, foi analisado apenas o joelho direito.

As mensurações das distâncias ML e AP no fêmur e tíbia foram realizadas a partir das imagens de RM por método validado na população brasileira em cortes axiais em sequência T2 no próprio software: 1

**Fig. 1:**
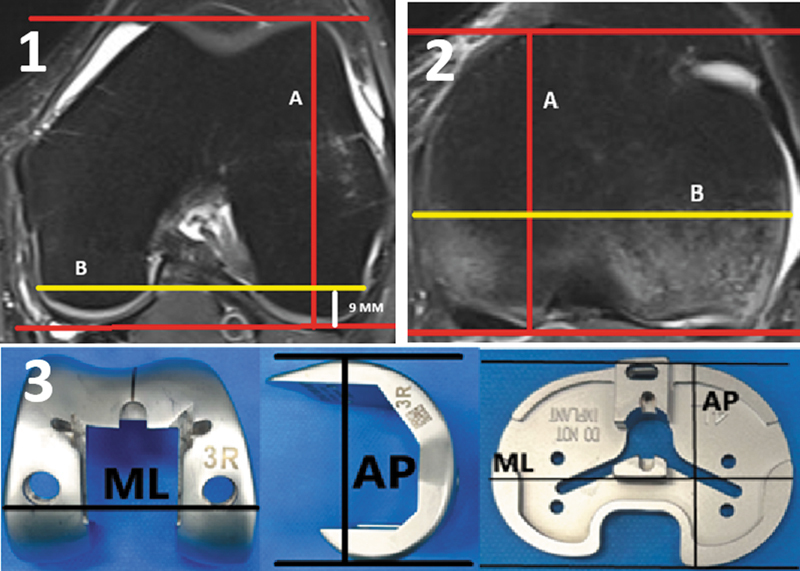
Medida anteroposterior (AP) ( **A** ), referenciada pela mais anterior e posterior proeminência articular do côndilo femoral lateral, e medida mediolateral (ML) bicortical ( **B** ), aferida a 9 mm da superfície articular posterior femoral, realizada no nível do corte axial epicondilar. Medida AP ( **C** ), referenciada pela mais anterior e posterior proeminência articular do planalto tibial, e medida ML ( **D** ), aferida no maior eixo bicortical da superfície articular tibial, no nível do corte axial da inserção do ligamento cruzado posterior (LCP). ( **E** ) Medidas AP e ML dos implantes femoral e tibial de artroplastias.

- Dimensão AP femoral: a maior distância de eixo AP avaliado no côndilo lateral entre as extremidades mais proeminentes articulares posterior e anterior ( Fig. 1A ).
- Dimensão ML femoral: distância do eixo bicortical a 9 mm da linha articular posterior do joelho ( Fig. 1B ).
- Dimensão AP tibial: no nível da inserção tibial do LCP foi utilizada o maior eixo perpendicular a cortical posterior do planalto tibial medial ( Fig. 1C ).
- Dimensão ML tibial: no nível da inserção tibial do ligamento cruzado posterior (LCP) foi utilizada a maior distância entre as corticais medial e lateral tibial paralelamente a cortical posterior ( Fig. 1D ).

Todas as mensurações foram realizadas por único cirurgião de joelho duas vezes e em dias diferentes. Foram computadas somente as mensurações que apresentaram variação menor que 10% entre na análise intraobservador.

A busca dos fabricantes internacionais de implantes de ATJ foi realizada no site da Instituição Orthopaedic Data Evaluation Panel (ODEP https://www.odep.org.uk/methodology/methodology-for-tkr/ ). Todos os fabricantes internacionais e nacionais foram contactados por e-mail. Também foram feitas buscas das informações em seus sites comerciais a fim de obter os dados associados às medidas ML e AP de seus implantes de ATJ. As mensurações corresponderam ao eixo de maior proeminência AP e ML dos implantes nos dois planos indicados pelos fabricantes ( Fig. 1E ).

Foram identificados 20 fabricantes e 25 implantes: Columbus (Aesculap AG), AKS (Baumer), Sigma e Attune (DePuy Synthes), ACS (Implantcast GmbH), NG e PAR (Impol Instrumental e Implantes Ltda.), Gemini e Symphoknee (Waldemar Link GmbH & Co.), Freedom (Maxx Orthopedics), Sphere (Medacta Corp.), Freedom (Meril Life), Advance (Microport Orthopedics Inc.), Orthovasive Indus (Biorad Medisys Pvt Ltd.), Ortosíntese (Ortosintese), BPK-S (Peter Brehm GmbH), Sartori (Sartori), Legion (Smith + Nephew), Scorpio e Triathlon (Stryker), U2 (United Orthopedic Corp.), MetaBio e Rotaflex (Vincula), além de Persona e Nex Gen (Zimmer Biomet).

### Análise Estatística

Dados epidemiológicos quanto ao sexo, idade e dimensões morfológicas foram analisados, comparados entre si, e expressos em termos de média e desvio padrão (DP).

Para determinar o implante femoral mais adequado de cada fabricante para cada paciente, priorizou-se a distância mais próxima AP, podendo ser igual, subdimensionada ou hiper dimensionada. A medida de referência ML foi usada paraidentificar os componentes tibiais mais adequados. As análises médias incluíram tanto valores de subcobertura quanto de sobrecobertura para a as medidas AP e ML.

As relações das dimensões do fêmur e tíbia dos pacientes e as mesmas dimensões das próteses foram relacionadas utilizando a distância euclidiana para comparar sua proximidade em um espaço bidimensional. A fórmula utilizada foi:



Foram analisadas as variações médias, máxima, DPs e diferença estatística (valor de *p* ) das relações entre as medidas anatômicas e dos implantes. Para verificar existência de diferenças significativas entre os tipos de próteses foi realizada a análise de variância (ANOVA). Os pressupostos de normalidade e homogeneidade de variâncias foram verificados pelos testes de Shapiro-Wilk e Levene. O nível de significância adotado foi de *p* = 0,05.

As medidas AP e ML dos implantes femorais e tibiais foram agregadas a fim de identificar o ranqueamento dos implantes que mais se customizaram aos joelhos dos pacientes. Sendo assim, os implantes menor variação entre os dispositivos e as morfologias ósseas receberam escores maiores. Para criar um ranqueamento dos implantes de próteses, foi utilizada a normalização dos valores para o intervalo de 0 a 1, seguida da criação de um escore (valor normalizado da tíbia e valor normalizado fêmur). O cálculo amostral foi determinado para um nível de confiança de 90%.

## Resultados

Entre 500 pacientes avaliados (250 homens e 250 mulheres) a idade média foi de 51 anos, com média de 53 anos entre as mulheres e 49 anos entre os homens ( Tabela 1 ).

**Tabela 1:** Dados epidemiológicos e análise estatística morfológica das distâncias femorais mediolateral (ML) e anteroposterior (AP)

	Total (média)	Sexo masculino (média)	Sexo feminino (média)
**Pacientes (n)**	500	250	250
**Idade (anos)**	50,91 ± 14,76	48,82 ± 15,43	53,01 ± 13,73
**ML: fêmur (mm)**	72,11 ± 5,93	76,22 ± 4,83	68,00 ± 3,65
**AP: fêmur (mm)**	67,34 ± 5,03	69,92 ± 5,14	64,74 ± 3,30
**ML: tíbia (mm)**	73,63 ± 6,48	76,89 ± 6,86	70,36 ± 3,94
**AP: tíbia (mm)**	53,02 ± 4,94	56,23 ± 4,45	49,81 ± 2,92

As médias das medidas dos fêmures apresentaram um dimensionamento AP de 69,92 ± 5,14 mm) e largura ML de 76,22 ± 4,83 mm entre os homens, e dimensionamento AP de 64,74 ± 3,30 mm e largura ML de 68,00 ± 3,65 mm entre as mulheres, como pode ser visto na Tabela 1 .

A média de divergência para a relação entre os implantes e os fêmures femininos nas relações AP/ML foi de 4,23 mm. As variações médias para as medidas AP foram de 0,98 mm, sendo de 1,11 mm para anatomias maiores que os implantes e de 0,91 mm para implantes maiores que a anatomia. Para as medidas ML a média foi de 3,94 mm, sendo a anatomia maior que os implantes 1,75 mm em média e para implantes maiores que a anatomia 4,15 mm. A maioria dos implantes apresentaram medidas ML maiores do que os fêmures. A melhor relação de medidas morfológicas foi encontrada no Sphere, com divergências médias ML/AP de 2,36 ± 1,66 mm ( Tabela 2 ).

**Tabela 2:** Comparação dos dados morfológicos dos fêmures dos pacientes femininos com os implantes para as medidas mediolaterais (ML) e anteroposteriores (AP)

	Distância euclidiana (AP e ML)					Medidas ML								Medidas AP							
Fabricante; pPrótese	Média (mm)	Mín (mm)	Máx (mm)	DP	Valor de *p*	Média (mm)	DP	Máx (mm)	Prótese < paciente (n)	Média Prótese < paciente (mm)	Prótese > paciente (n)	Média Prótese > paciente (mm)	Prótese = paciente (n)	Média (mm)	DP	Máx (mm)	Prótese < paciente (n)	Média Prótese < paciente (mm)	Prótese > paciente (n)	Média Prótese > paciente (mm)	Prótese = paciente (n)
Medacta; Sphere	2,36	0,20	8,45	1,66		0,46	0,55	1,00	116	0,54	114	0,46	20	2,25	2,82	8,4	133	2,38	112	2,19	5
Unites Orthopedic; U2	2,36	0,00	8,34	1,69	1,000	0,54	0,61	1,00	128	0,60	108	0,54	14	2,22	2,76	8,3	96	1,99	149	2,43	5
Link; Symphoknee	2,39	0,14	7,30	1,41	1,000	0,79	0,90	1,50	123	0,82	117	0,83	10	2,11	2,41	7,3	105	1,29	142	2,77	3
Aesculap; Columbus	2,39	0,00	7,28	1,39	1,000	1,11	1,30	2,50	138	1,20	102	1,10	10	1,91	2,24	7	174	2,05	72	1,69	4
Implantcast; ACS Knee System	2,53	0,10	7,45	1,44	1,000	0,95	1,14	3,50	113	1,11	126	0,89	11	2,18	2,10	7,4	206	2,34	40	1,58	4
Link; Gemini	2,72	0,28	10,30	1,88	0,989	0,83	1,00	3,00	132	0,93	109	0,77	9	2,42	2,67	10,3	71	1,32	176	2,91	3
Maxx Orthopedics; Freedom	2,76	0,00	7,45	1,65	0,969	0,85	0,99	2,00	140	0,91	101	0,84	9	2,46	3,00	7,4	104	2,25	141	2,71	5
Meril; Freedom	2,79	0,00	7,45	1,67	0,930	0,85	0,99	2,00	138	0,91	102	0,84	10	2,49	3,03	7,4	104	2,25	141	2,76	5
DePuy; Attune	3,09	0,32	10,20	1,87	0,050	0,78	0,91	1,60	109	0,83	133	0,79	8	2,86	2,83	10,1	55	1,83	192	3,20	3
Microport; Advance	3,36	0,22	11,44	1,96	0,000	1,00	1,14	2,00	120	1,05	121	1,02	9	3,08	2,83	11,3	43	1,95	205	3,35	2
DePuy; Sigma	3,45	0,32	11,44	1,77	0,000	1,09	1,38	6,00	128	1,31	110	0,97	12	3,06	3,16	11,3	71	2,29	176	3,42	3
Zimmer; Persona	3,47	0,36	9,30	1,97	0,000	0,83	0,94	1,90	121	0,87	120	0,85	9	3,26	2,80	9,3	39	1,84	210	3,54	0
Stryker; Triathlon	3,49	0,36	9,30	2,01	0,000	0,79	0,90	1,50	121	0,84	119	0,81	10	3,30	2,83	9,3	39	1,84	210	3,58	1
Orthovasive; Indus	3,61	0,36	9,30	2,29	0,000	1,44	1,88	8,00	122	1,72	126	1,19	2	3,05	3,26	9,3	63	2,05	185	3,42	2
Smith & Nephew; Legion	3,97	0,22	11,30	2,18	0,000	0,67	0,81	2,00	129	0,78	105	0,63	16	3,83	3,01	11,3	34	2,04	215	4,13	1
Stryker; Scorpio	4,06	0,20	11,33	2,21	0,000	0,93	1,21	5,00	137	1,15	98	0,77	15	3,77	3,05	11,3	35	1,92	214	4,10	1
Peter Brehm; BPK-S	4,22	0,73	12,26	2,18	0,000	1,43	1,60	3,00	149	1,46	95	1,46	6	3,77	3,28	12,1	39	2,37	210	4,05	1
Vincula; Rotaflex	4,31	0,22	11,60	2,55	0,000	0,44	0,52	1,50	133	0,53	95	0,42	22	4,24	3,31	11,6	34	1,92	215	4,63	1
Impol; PAR	4,88	1,08	12,61	2,09	0,000	1,68	2,14	7,00	114	2,01	128	1,49	8	4,25	2,76	12,3	19	1,69	228	4,52	3
Impol; NG	5,99	0,94	13,32	2,79	0,000	1,14	1,50	7,00	131	1,43	112	0,87	7	5,68	3,48	13,3	17	2,04	232	5,97	1
Zimmer; Nex Gen	6,66	0,36	13,30	2,77	0,000	1,44	1,92	9,00	146	1,80	100	0,96	4	6,21	3,27	13,3	10	1,49	239	6,44	1
Sartori; Sartori	7,14	0,36	13,30	3,09	0,000	1,07	1,28	5,00	129	1,18	117	0,97	4	6,95	3,27	13,3	3	0,70	247	7,03	0
Baumer; Baumer	7,56	1,25	14,32	3,07	0,000	1,05	1,31	5,00	118	1,13	125	1,04	7	7,40	3,24	14,3	3	1,23	247	7,48	0
Vincula; MetaBio	7,73	0,14	13,50	3,05	0,000	1,10	1,30	4,50	133	1,24	111	0,99	6	7,58	3,16	13,4	2	0,30	248	7,64	0
Ortosintese; Ortosintese	8,35	1,08	17,95	2,94	0,000	1,28	1,51	4,00	145	1,40	93	1,28	12	8,15	3,09	17,8	2	0,30	248	8,22	0

Abreviações: DP, desvio padrão; Max, valor máximo; Min, valor mínimo.

A média de divergência para os fêmures masculinos nas medidas AP/ML foi de 4,71 mm. As variações médias para as medidas AP foram de 1,94 mm, sendo 2,39 mm para anatomias maiores que os implantes e 0,92 mm com implantes maiores que a anatomia. Para as medidas ML a média de divergência foi de 3,81 mm, sendo a anatomia maior que os implantes em média 4,02 mm e os implantes maiores que a anatomia 2,87 mm. A maioria dos implantes apresentaram medidas ML menores que as anatomias. A melhor relação de medidas morfológicas foi encontrada no Triathlon, com divergências médias de relação ML/AP de 3,52 ± 2,69 mm ( Tabela 3 ).

**Tabela 3:** Comparação dos dados morfológicos dos fêmures dos pacientes masculinos com os implantes para as medidas mediolaterais (ML) e anteroposteriores (AP)

	Distância euclidiana (AP e ML)					Medidas ML								Medidas AP							
Fabricante; prótese	Média (mm)	Mín (mm)	Máx (mm)	DP	Valor de *p*	Média (mm)	DP	Máx (mm)	Prótese < paciente (n)	Média prótese < paciente (mm)	Prótese > paciente (n)	Média Prótese > paciente (mm)	Prótese = paciente (n)	Média (mm)	DP	Máx (mm)	Prótese < paciente (n)	Média Prótese < paciente (mm)	Prótese > paciente (n)	Média Prótese > paciente (mm)	ML prótese = ML paciente (n)
**Stryker; Triathlon**	**3,52**	**0,14**	**13,61**	**2,69**		**1,00**	**1,67**	**10,00**	**143**	**1,28**	**99**	**0,68**	**8**	**3,16**	**3,94**	**13,6**	**145**	**3,62**	**102**	**2,59**	**3**
Zimmer;Persona	3,55	0,14	13,61	2,68	1,000	1,15	1,77	10,00	141	1,39	102	0,90	7	3,12	3,89	13,6	150	3,60	97	2,48	3
Microport; Advance	3,63	0,41	15,22	2,50	1,000	1,29	1,77	9,00	102	1,63	143	1,10	5	3,19	3,79	15,1	158	3,61	92	2,47	0
Link; Symphoknee	3,64	0,36	12,61	2,61	1,000	0,92	1,37	8,00	138	1,09	106	0,76	6	3,33	3,59	12,6	177	3,95	70	1,88	3
DePuy; Attune	3,65	0,14	15,16	2,81	1,000	1,02	1,41	7,70	136	1,16	109	0,89	5	3,32	4,12	15,1	141	4,26	108	2,13	1
Smith & Nephew; Legion	3,68	0,10	14,61	2,47	1,000	1,02	1,68	10,00	156	1,24	84	0,73	10	3,34	4,05	14,6	122	3,74	128	2,96	0
Aesculap; Columbus	3,73	0,14	15,22	2,54	1,000	1,11	1,33	4,50	137	1,20	111	1,01	2	3,39	3,72	15,1	196	3,55	54	2,79	0
Meril; Freedom	4,03	0,36	15,61	3,27	0,993	1,04	1,70	10,00	152	1,27	89	0,77	9	3,61	4,42	15,6	160	4,42	87	2,24	3
United Orthopedic; U2	4,08	0,36	15,61	3,34	0,974	1,05	1,83	11,00	152	1,42	87	0,54	11	3,72	3,90	15,6	192	4,35	56	1,67	2
Implantcast; ACS Knee System	4,10	0,32	16,66	2,86	0,961	1,39	1,61	6,00	168	1,54	76	1,16	6	3,60	3,47	16,6	224	3,77	25	2,27	1
Peter Brehm; BPK-S	4,15	0,22	15,46	2,37	0,906	1,43	1,88	8,50	114	1,64	133	1,29	3	3,63	4,36	15,3	102	3,75	146	3,60	2
Medacta; Sphere	4,19	0,22	17,12	3,43	0,839	0,67	1,23	8,00	119	0,89	116	0,53	15	4,02	3,97	17,1	207	4,53	40	1,71	3
Maxx Orthopedics; Freedom	4,21	0,36	15,61	3,42	0,800	1,27	1,92	11,00	164	1,54	79	0,83	7	3,76	3,99	15,6	189	4,50	57	1,56	4
Vincula; Rotaflex	4,24	0,42	13,61	2,85	0,731	1,05	1,79	11,00	172	1,36	62	0,44	16	3,78	4,51	13,6	83	3,62	165	3,91	2
Link; Gemini	4,64	0,20	17,56	3,08	0,027	2,01	2,70	13,00	148	2,65	96	1,14	6	3,81	3,85	12,6	176	4,65	71	1,87	3
Stryker; Scorpio	4,85	0,10	18,48	3,29	0,001	2,49	3,25	15,00	174	3,20	73	0,90	3	3,59	4,26	14,1	147	4,17	102	2,80	1
Sartori; Sartori	5,24	0,10	17,39	2,86	0,000	2,42	3,22	15,00	175	3,14	68	0,79	7	3,87	4,44	10,1	98	3,31	151	4,26	1
Ortosintese; Ortosintese	5,49	0,14	15,56	2,92	0,000	2,47	3,10	14,00	153	3,25	95	1,26	2	4,21	4,29	11,6	64	2,58	185	4,80	1
Vincula; MetaBio	5,54	0,10	16,02	2,91	0,000	2,36	3,11	14,50	170	3,03	75	1,01	5	4,22	4,38	11,6	69	2,80	180	4,79	1
Impol; PAR	5,64	0,78	20,69	3,71	0,000	3,68	4,09	17,00	192	4,28	56	1,76	2	3,67	4,21	11,8	155	4,28	94	2,71	1
Baumer; Baumer	5,70	0,28	16,47	2,98	0,000	2,42	3,31	15,00	157	3,37	90	0,83	3	4,27	4,41	11,4	74	2,52	175	5,03	1
Impol; NG	5,92	0,22	20,69	3,64	0,000	3,43	3,88	17,00	186	4,34	58	0,86	6	4,08	4,82	11,8	137	4,52	113	3,54	0
DePuy; Sigma	6,21	0,36	21,80	4,51	0,000	2,95	3,48	16,00	199	3,48	44	1,00	7	5,06	4,54	15,6	209	5,67	39	2,09	2
Zimmer; Nex Gen	6,80	0,10	22,37	3,95	0,000	4,73	4,30	19,00	217	5,36	27	0,71	6	3,91	4,65	11,8	131	4,57	119	3,19	0
Orthovasive; Indus	7,30	0,20	23,95	4,84	0,000	4,13	4,16	18,00	199	4,94	46	1,04	5	5,54	4,65	15,8	212	6,14	36	2,30	2

Abreviações: DP, desvio padrão; Max, valor máximo; Min, valor mínimo.

Os resultados dos achados morfológicos tibiais no sexo feminino demonstraram que os componentes apresentaram divergências médias ML/AP de 3,63 mm. O implante Legion apresentou a menor variação média de 0,88 mm de divergência ML para a anatomia. O quase metade das mulheres apresentaram implantes hiperdimensionados em 1,33 mm e subdimensionados 1,26 mm, em média, para a medida ML tibial. A relação AP média demonstrou divergência para as medidas anatômicas de 3,14 mm. A média de implantes com hiperdimensionamento AP foi de 0,85 mm e com sub- de 3,23 mm. Destaca-se o implante Advance, com 202 mulheres apresentando hiperdimensionamento AP, com média de 3,46 mm ( Tabela 4 ).

**Tabela 4:** Comparação dos dados morfológicos das tíbias dos pacientes femininos com os implantes para as medidas mediolaterais (ML) e anteroposteriores (AP)

	Distância euclidiana (AP e ML)					Medidas ML								Medidas AP							
Fabricante; prótese	Média (mm)	Mín (mm)	Máx (mm)	DP	Valor de *p*	Média (mm)	DP	Máx (mm)	Prótese < paciente (n)	Média prótese < paciente (mm)	Prótese > paciente (n)	Média prótese > paciente (mm)	Prótese = paciente (n)	Média (mm)	DP	Máx (mm)	Prótese < paciente (n)	Média prótese < paciente (mm)	AP prótese > paciente (n)	Média prótese > paciente (mm)	Prótese = paciente (n)
Smith & Nephew; Legion	2,19	0,22	17,76	1,55		0,88	1,37	15,8	129	0,82	117	0,97	4	1,85	2,29	13,20	130	2,03	119	1,67	1
Zimmer; Persona	2,54	0,20	16,74	1,61	0,984	1,09	1,46	13,5	130	1,12	115	1,11	5	2,14	2,62	14,00	124	2,28	123	2,04	3
United Orthopedic; U2	2,66	0,10	20,47	2,02	0,740	0,88	1,52	18,8	131	0,88	111	0,96	8	2,34	2,38	14,20	192	2,67	53	1,33	5
Aesculap; Columbus	2,74	0,58	18,82	1,51	0,395	1,37	1,86	17,8	113	1,42	132	1,38	5	2,12	1,37	12,20	246	2,15	3	0,27	1
Maxx Orthopedics; Freedom	2,76	0,14	17,92	1,83	0,330	1,29	1,74	14,8	127	1,23	120	1,38	3	2,19	2,40	13,20	172	2,66	74	1,22	4
Vincula; Rotaflex	2,83	0,14	15,52	1,66	0,134	1,56	1,94	13,8	101	1,63	147	1,54	2	2,11	2,32	13,20	173	2,42	76	1,42	1
Meril; Freedom	2,92	0,20	17,92	1,80	0,029	1,29	1,74	14,8	127	1,23	120	1,38	3	2,39	2,36	13,20	191	2,72	57	1,36	2
Zimmer; Nex Gen	3,31	0,20	15,27	2,20	0,000	1,03	1,46	11,8	111	1,11	132	1,02	7	2,98	2,57	15,20	211	3,34	37	1,11	2
Microport; Advance	3,37	0,20	14,07	1,89	0,000	1,07	1,31	9,8	112	1,03	134	1,13	4	3,03	2,57	10,10	47	1,25	202	3,46	1
Stryker; Triathlon	3,63	0,32	19,60	2,31	0,000	0,93	1,45	16,8	110	0,88	133	1,02	7	3,36	2,41	15,20	235	3,52	15	0,96	0
Orthovasive; Indus	3,63	0,10	17,99	2,41	0,000	1,20	1,84	15,8	121	1,35	123	1,11	6	3,21	2,46	14,20	222	3,55	24	0,62	4
Baumer; Baumer	3,66	0,28	17,10	1,86	0,000	1,92	2,32	13,8	144	1,88	103	2,03	3	2,79	1,90	15,20	250	2,79	0	0,00	0
Impol; NG	3,68	0,20	23,17	2,31	0,000	2,00	2,72	22,8	133	1,99	113	2,07	4	2,73	2,03	15,20	244	2,78	6	0,42	0
Sartori; Sartori	3,68	0,20	19,99	2,04	0,000	1,62	2,12	17,8	146	1,65	100	1,63	4	3,05	1,96	15,20	250	3,05	0	0,00	0
Vincula; MetaBio	3,70	0,20	19,99	2,06	0,000	1,62	2,11	17,8	147	1,67	99	1,61	4	3,07	1,98	15,20	250	3,07	0	0,00	0
Stryker; Scorpio	3,71	0,30	19,60	2,20	0,000	1,10	1,65	16,8	141	1,05	105	1,20	4	3,35	2,29	16,20	236	3,50	12	0,93	2
Ortosintese; Ortosintese	3,80	0,28	15,53	1,84	0,000	1,98	2,33	11,8	146	2,01	102	1,98	2	2,92	1,93	15,20	250	2,92	0	0,00	0
DePuy; Attune	3,81	0,14	18,50	2,24	0,000	0,85	1,30	14,8	121	0,79	123	0,94	6	3,60	2,26	16,50	241	3,72	9	0,47	0
Medacta; Sphere	3,81	0,58	18,75	2,15	0,000	1,38	1,79	15,8	105	1,39	140	1,41	5	3,34	2,39	15,20	230	3,55	18	1,03	2
DePuy; Sigma	3,85	0,10	18,66	2,29	0,000	1,05	1,54	15	112	1,04	132	1,09	6	3,54	2,44	15,40	236	3,69	12	1,21	2
Link; Gemini	3,95	0,22	19,56	2,39	0,000	1,35	1,87	17,8	98	1,28	146	1,45	6	3,44	2,58	15,20	226	3,74	22	0,64	2
Link; Symphoknee	4,35	0,20	19,44	2,37	0,000	0,92	1,36	14,8	115	0,90	128	0,99	7	4,15	2,36	16,20	243	4,26	6	0,42	1
Impol; PAR	4,41	0,22	22,39	2,54	0,000	1,38	2,04	21,8	120	1,26	128	1,52	2	3,99	2,46	16,20	240	4,14	9	0,37	1
Implantcast; ACS Knee System	4,83	0,95	19,90	2,38	0,000	1,04	1,55	15,8	123	1,04	119	1,11	8	4,59	2,35	17,70	246	4,66	3	0,27	1
Peter Brehm; BPK-S	6,35	2,13	20,59	2,48	0,000	1,06	1,51	15,8	113	0,95	130	1,21	7	6,19	2,41	17,90	250	6,19	0	0,00	0

Abreviações: DP, desvio padrão; Max, valor máximo; Min, valor mínimo.

Na população masculina, os valores de ML e AP tibiais demonstraram que o implante com a melhor relação de medidas morfológicas foi o Persona, com divergências médias de relação ML/AP de 3,27 ± 3,00 mm. O número médio de implantes hiperdimensionados ML foi de 1,77 mm, e de subdimensionados, de 1,99 mm. A relação AP, em média, demonstrou divergência para as medidas anatômicas de 5,54 mm. A média geral de implantes com hiperdimensionamento AP foi de 0,69 mm e a de sub foi 5,78 mm ( Tabela 5 ).

**Tabela 5:** Comparação dos dados morfológicos das tíbias dos pacientes masculinos com os implantes para as medidas mediolaterais (ML) e anteroposteriores (AP)

	Distância euclidiana ( AP e ML)					Medidas ML								Medidas AP							
Fabricante; prótese	Média (mm)	Mín (mm)	Máx (mm)	DP	*p*	Média (mm)	DP	Máx (mm)	ML prótese < ML paciente (n)	Média ML prótese < ML paciente (mm)	ML prótese > ML paciente (n)	Média ML prótese > ML paciente (mm)	ML prótese = ML paciente (n)	Média (mm)	DP	Máx (mm)	AP prótese < AP paciente (n)	Média AP prótese < AP paciente (mm)	AP prótese > AP paciente (n)	Média AP prótese > AP paciente (mm)	AP prótese = AP paciente (n)
Zimmer;Persona	3,27	0,14	17,18	3,00		1,14	1,57	8,2	115	1,01	130	1,29	5	2,90	3,97	15,60	134	3,83	116	1,82	0
Microport; Advance	3,38	0,22	16,18	2,53	1,000	1,03	1,26	4,5	125	1,04	119	1,08	6	3,05	3,90	15,80	67	3,79	181	2,81	2
Smith & Nephew; Legion	3,50	0,10	16,76	3,17	1,000	1,12	1,79	10,5	124	0,93	119	1,39	7	3,19	3,62	13,80	194	3,63	54	1,72	2
Aesculap; Columbus	4,34	0,36	16,55	3,17	0,233	1,43	2,21	12,5	100	1,27	143	1,62	7	3,90	2,94	12,90	250	3,90	0	0,00	0
Vincula; Rotaflex	4,36	0,32	14,84	3,18	0,202	1,40	1,86	8,5	113	1,33	132	1,51	5	3,89	3,51	12,80	220	4,28	27	1,08	3
Stryker; Triathlon	4,39	0,51	18,97	3,53	0,157	1,19	1,96	11,5	124	1,02	120	1,42	6	4,07	3,91	15,80	204	4,67	46	1,42	0
United Orthopedic; U2	4,72	0,42	18,68	3,73	0,006	2,06	3,04	13,5	138	2,35	110	1,75	2	3,97	3,78	13,80	204	4,59	43	1,32	3
Medacata.; Sphere	5,28	0,50	18,44	3,58	0,000	1,57	2,11	10,5	97	1,34	148	1,77	5	4,80	3,70	15,80	236	5,05	11	0,86	3
Meril; Freedom	5,49	0,32	17,94	4,19	0,000	2,58	3,21	9,9	164	3,16	82	1,53	4	4,52	4,06	15,80	219	5,03	28	1,01	3
Maxx Orthopedics; Freedom	5,49	0,32	17,94	4,19	0,000	2,58	3,21	9,9	164	3,16	82	1,53	4	4,52	4,06	15,80	219	5,03	28	1,01	3
DePuy; Sigma	5,74	0,89	18,92	3,60	0,000	1,53	2,00	9,7	102	1,30	145	1,72	3	5,33	3,70	16,80	242	5,48	8	0,70	0
Link; Gemini	5,75	0,94	18,01	3,41	0,000	1,64	2,44	12,5	128	1,43	115	1,97	7	5,26	3,36	13,80	243	5,40	6	0,67	1
Stryker; Scorpio	5,77	0,10	18,97	3,53	0,000	1,72	2,33	11,5	116	1,52	130	1,96	4	5,26	3,57	15,80	240	5,47	7	0,40	3
DePuy.; Attune	5,94	0,22	18,83	3,54	0,000	0,94	1,54	9,5	123	0,77	121	1,16	6	5,79	3,45	16,80	247	5,86	3	0,20	0
Link; Symphoknee	6,18	0,64	20,18	3,77	0,000	1,18	1,77	9,5	132	1,16	111	1,29	7	5,98	3,68	18,30	247	6,06	1	0,20	2
Ortosintese; Ortosintese	6,44	1,04	16,73	3,49	0,000	2,04	2,46	6,5	128	2,04	120	2,07	2	5,89	3,59	15,80	250	5,89	0	0,00	0
Implantcast; ACS Knee System	6,84	1,62	20,18	3,69	0,000	1,49	2,01	10,5	99	1,20	145	1,74	6	6,54	3,65	17,80	250	6,54	0	0,00	0
Sartori; Sartori	7,10	0,22	18,77	4,02	0,000	2,18	2,89	12,5	125	1,99	123	2,42	2	6,56	3,89	17,40	250	6,56	0	0,00	0
Baumer; Baumer	7,20	0,54	17,68	3,98	0,000	2,05	2,54	8,5	117	2,13	128	2,06	5	6,71	4,03	17,60	250	6,71	0	0,00	0
Vincula; MetaBio	7,58	0,22	18,77	4,23	0,000	2,18	2,90	12,5	128	2,03	120	2,38	2	7,07	4,12	18,40	250	7,07	0	0,00	0
Zimmer; Nex Gen	7,90	0,22	20,70	4,66	0,000	2,93	3,24	10,9	193	3,41	54	1,38	3	7,14	4,27	18,40	244	7,28	5	1,60	1
Impol; NG	8,61	0,94	21,82	4,95	0,000	4,69	5,21	17,5	181	5,27	67	3,27	2	6,90	3,90	18,40	250	6,90	0	0,00	0
Peter Brehm; BPK-S	8,72	2,62	21,15	3,54	0,000	1,24	1,87	10,5	125	1,02	116	1,58	9	8,57	3,41	18,90	250	8,57	0	0,00	0
Orthovasive; Indus	9,22	0,78	22,64	4,97	0,000	4,23	4,34	12,9	184	5,10	63	1,89	3	7,92	4,22	19,40	243	8,14	6	0,45	1
Impol; PAR	9,37	0,99	21,74	4,60	0,000	2,62	3,81	16,5	171	2,76	77	2,37	2	8,75	4,08	20,40	250	8,75	0	0,00	0

**Fig. 2:**
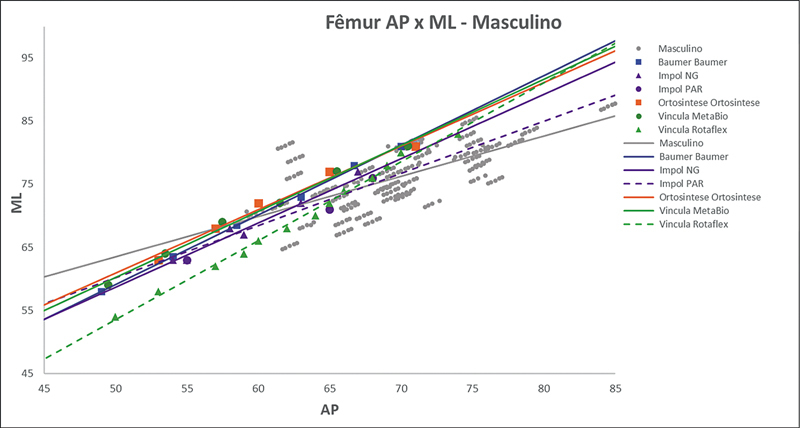
Linhas de regressão ML *versus* AP das medidas do fêmur em homens e implantes Baumer, NG, PAR, Ortosintese, MetaBio e Rotaflex.

As Figs. 2 3 4 5 6 7 8 9 de regressões demonstram os agrupamentos dos implantes nacionais e importados líderes do mercado mundial em ATJ, 15 quanto às medidas ML e AP femorais e tibiais. Os resultados demonstram que os implantes importados se comportam com melhor “customização” morfológica, principalmente nos joelhos masculinos. As maiores divergências se apresentaram para os fêmures femininos.

**Fig. 3:**
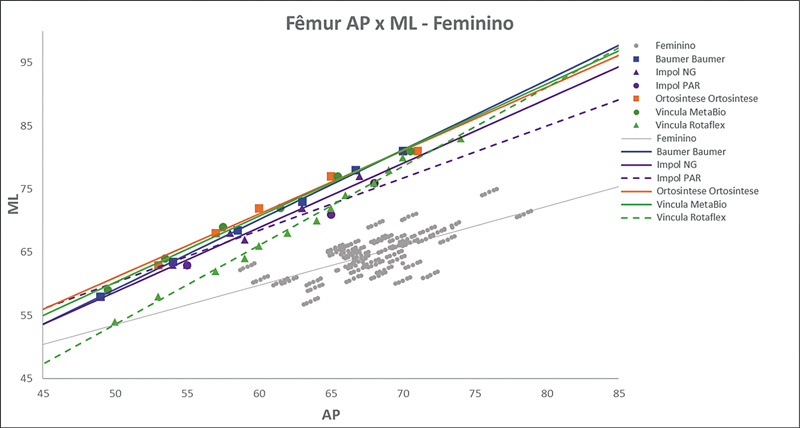
Linhas de regressão ML *versus* AP das medidas do fêmur em mulheres e implantes Baumer, NG, PAR, Ortosintese, MetaBio e Rotaflex.

**Fig. 4:**
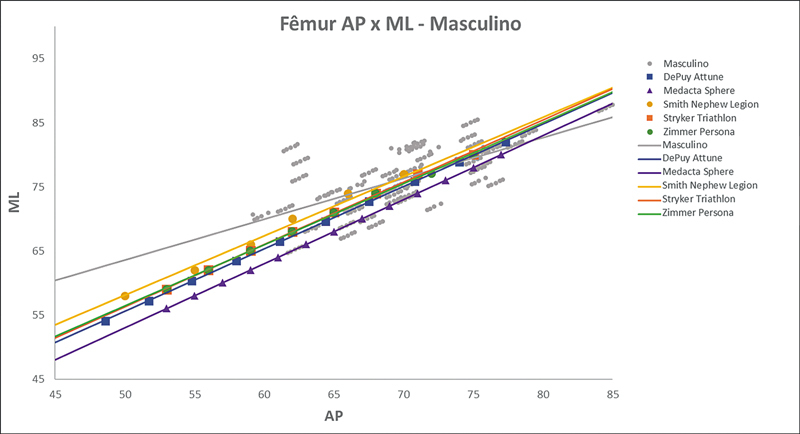
Linhas de regressão ML *versus* AP das medidas do fêmur em homens e implantes Attune, Sphere, Legion, Triathlon e Persona.

**Fig. 5:**
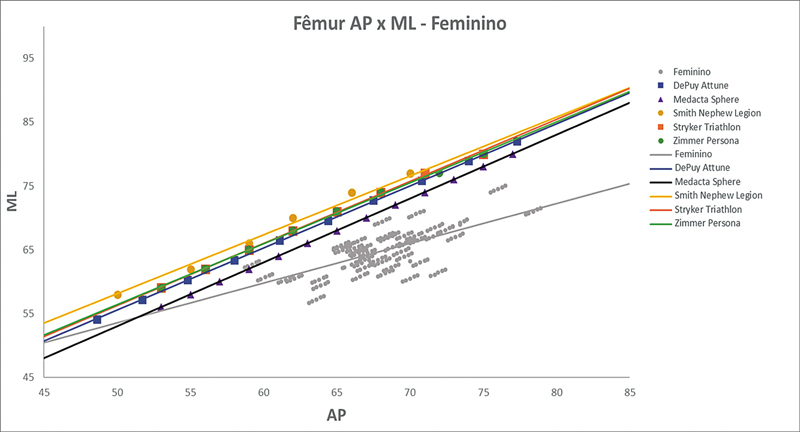
Linhas de regressão ML *versus* AP das medidas do fêmur em mulheres e implantes Attune, Sphere, Legion, Triathlon e Persona.

**Fig. 6:**
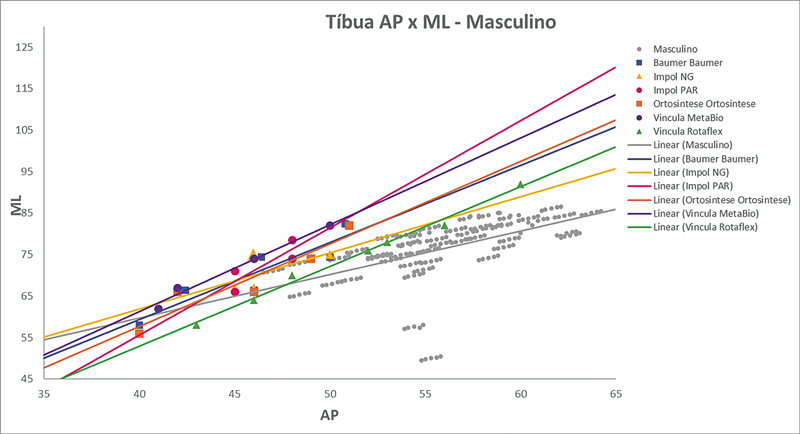
Linhas de regressão ML *versus* AP das medidas da tíbia em homens e implantes Baumer, NG, PAR, Ortosintese, MetaBio e Rotaflex.

**Fig. 7:**
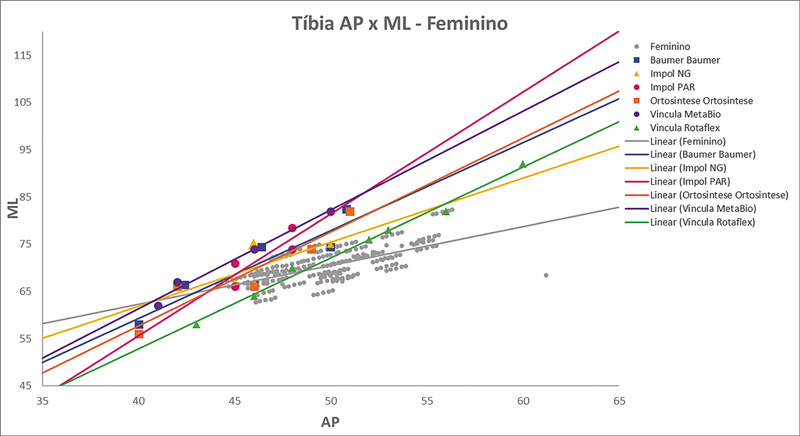
Linhas de regressão ML *versus* AP das medidas da tíbia em mulheres e implantes Baumer, NG, PAR, Ortosintese, MetaBio e Rotaflex.

**Fig. 8:**
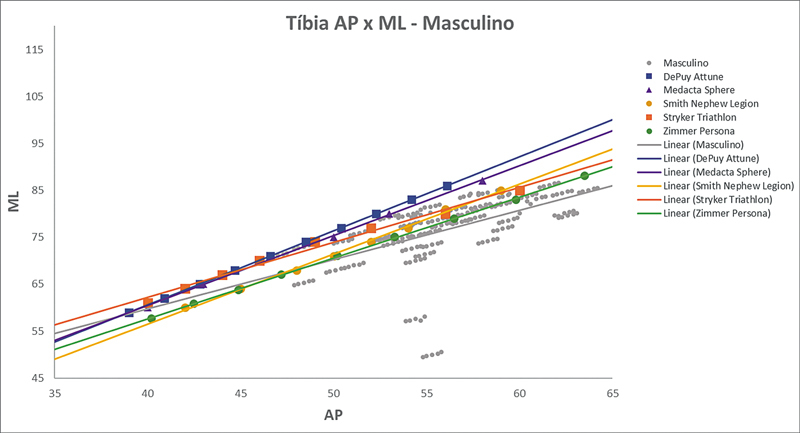
Linhas de regressão ML *versus* AP das medidas da tíbia em homens e implantes Attune, Sphere, Legion, Triathlon e Persona.

**Fig. 9:**
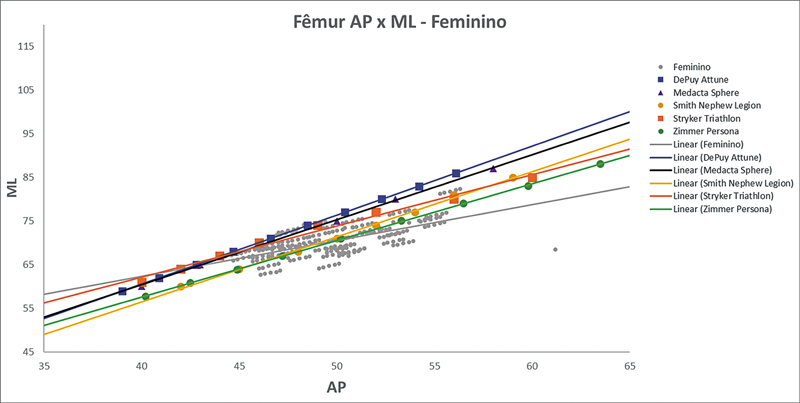
Linhas de regressão ML *versus* AP das medidas da tíbia em mulheres e implantes Attune, Sphere, Legion, Triathlon e Persona.

Em mulheres, considerando a acomodação AP/ML para implantes de ATJ, observou-se que o implante U2 apresentou a melhor pontuação estatística, seguido dos implantes Columbus e Legion ( Tabela 6 ). Para os homens, os melhores foram Persona, Advance e Legion ( Tabela 7 ).

**Tabela 6:** Ranking dos implantes para a acomodação dos implantes nos pacientes do sexo feminino

		Valores utilizados para calcular o Escore Final					
		Valores tíbia			Valores fêmur		
Fabricante; prótese	Escore final	Valor da distância euclidiana	Ranking	Valor normalizado	Valor da distância euclidiana	Ranking	Valor normalizado
United Orthopedic; U2	0,11	2,66	3	0,11	2,36	2	0,00
Aesculap; Columbus	0,14	2,74	4	0,13	2,39	4	0,00
Smith & Nephew; Legion	0,27	2,19	1	0,00	3,97	16	0,27
Maxx Orthopedics; Freedom	0,20	2,76	5	0,14	2,76	7	0,07
Zimmer; Persona	0,27	2,54	2	0,08	3,47	13	0,19
Meril; Freedom	0,25	2,92	7	0,17	2,79	8	0,07
Vincula; Rotaflex	0,48	2,83	6	0,15	4,31	19	0,33
Microport; Advance	0,45	3,37	9	0,28	3,36	11	0,17
Medacta; Sphere	0,39	3,81	19	0,39	2,36	1	0,00
Link; Gemini	0,48	3,95	21	0,42	2,72	6	0,06
Stryker; Triathlon	0,53	3,63	10	0,35	3,49	14	0,19
DePuy; Attune	0,51	3,81	18	0,39	3,09	9	0,12
Orthovasive; Indus	0,55	3,63	11	0,35	3,61	15	0,21
DePuy; Sigma	0,58	3,85	20	0,40	3,45	12	0,18
Link; Symphoknee	0,52	4,35	23	0,52	2,39	3	0,00
Stryker; Scorpio	0,65	3,71	16	0,37	4,06	17	0,28
Implantcast; ACS Knee System	0,66	4,83	25	0,63	2,53	5	0,03
Zimmer; Nex Gen	0,99	3,31	8	0,27	6,66	22	0,72
Impol; NG	0,96	3,68	13	0,36	5,99	21	0,61
Impol; PAR	0,96	4,41	24	0,53	4,88	20	0,42
Sartori; Sartori	1,16	3,68	14	0,36	7,14	23	0,80
Baumer; Baumer	1,22	3,66	12	0,35	7,56	24	0,87
Vincula; MetaBio	1,26	3,70	15	0,36	7,73	25	0,90
Ortosintese; Ortosintese	1,39	3,80	17	0,39	8,35	26	1,00
Peter Brehm; BPK-S	1,31	6,35	26	1,00	4,22	18	0,31

**Tabela 7:** Ranking dos implantes para a acomodação dos implantes nos pacientes do sexo masculino

		Valores utilizados para calcular o escore final					
		Valores para a tíbia			Valores para o fêmur		
Fabricante; prótese	Escore final	Valor da distância euclidiana	Ranking	Valor normalizado	Valor da distância euclidiana	Ranking	Valor normalizado
Zimmer; Persona	0,01	3,27	1	0,00	3,55	2	0,01
Microport; Advance	0,05	3,38	2	0,02	3,63	3	0,03
Smith & Nephew; Legion	0,08	3,50	3	0,04	3,68	6	0,04
Aesculap; Columbus	0,23	4,34	4	0,18	3,73	7	0,06
Stryker; Triathlon	0,18	4,39	6	0,18	3,52	1	0,00
Vincula; Rotaflex	0,37	4,36	5	0,18	4,24	14	0,19
United Orthopedic; U2	0,39	4,72	7	0,24	4,08	9	0,15
Medacta; Sphere	0,51	5,28	8	0,33	4,19	12	0,18
Meril; Freedom	0,50	5,49	9	0,36	4,03	8	0,14
Maxx Orthopedics; Freedom	0,55	5,49	10	0,36	4,21	13	0,18
DePuy; Attune	0,47	5,94	14	0,44	3,65	5	0,04
Link; Symphoknee	0,51	6,18	15	0,48	3,64	4	0,03
Link; Gemini	0,70	5,75	12	0,41	4,64	15	0,30
Stryker; Scorpio	0,76	5,77	13	0,41	4,85	16	0,35
Implantcast; ACS Knee System	0,74	6,84	18	0,59	4,10	10	0,15
DePuy; Sigma	1,12	5,74	11	0,41	6,21	23	0,71
Ortosintese; Ortosintese	1,04	6,44	17	0,52	5,49	18	0,52
Sartori; Sartori	1,08	7,10	19	0,63	5,24	17	0,46
Peter Brehm; BPK-S	1,06	8,72	24	0,89	4,15	11	0,17
Baumer; Baumer	1,22	7,20	20	0,64	5,70	21	0,58
Vincula; MetaBio	1,24	7,58	21	0,71	5,54	19	0,53
Impol; NG	1,51	8,61	23	0,88	5,92	22	0,63
Impol; PAR	1,56	9,37	26	1,00	5,64	20	0,56
Zimmer; Nex Gen	1,63	7,90	22	0,76	6,80	24	0,87
Orthovasive; Indus	1,98	9,22	25	0,98	7,30	25	1,00

## Discussão

Os fabricantes de ATJ comumente utilizam grupos populacionais específicos como referências para o desenvolvimento de seus implantes. Apesar da diversidade étnica em suas amostras, é possível que populações apresentem características próprias de joelhos, o que contribuiria para divergências se comparados a outros grupos étnicos. 10 13 16

Os resultados encontrados demonstram que, em média, as variações morfológicas encontradas entre os implantes e joelhos dos brasileiros não exercem ampla preocupação para cirurgiões em relação aos dimensionamentos bidimensionais femorais e tibiais. No entanto foram encontradas pessoas de ambos os sexos, e em múltiplos implantes, com diferenças maiores que 10mm. Nestes casos, o cirurgião deve considerar modificar os dimensionamentos dos implantes para melhor acomodação anatômica. Essas diferenças influenciam diretamente nas condições biomecânicas do joelho, podendo interferir nos espaços articulares de extensão e flexão assim como na capacidade de intercambiar numerações diferentes de implantes femorais e tibiais, principalmente em dispositivos com ressecção do LCP em determinados fabricantes de implantes.

Destaca-se a importância biomecânica da medida AP para o implante femoral devido a sua influência na estabilidade do joelho e correspondência direta com o dimensionamento do espaço de flexão. O aumento ou diminuição do *offset* posterior no componente femoral pode alterar o espaço de flexão diretamente, assim como influenciar o espaço de extensão. Isso ocorre dada a relação do contato capsular e de partes moles posteriores do joelho, que pode contribuir para a diminuição ou aumento do espaço de extensão. 17 18 Os resultados do presente estudo demonstram que, em mulheres, nenhum implante femoral apresentou variações máximas de divergências médias maiores que 3 mm para a medida AP. No entanto, quatro implantes apresentaram média com divergências maiores que 3 mm em homens (PAR e NG, Nex Gen, Indus). Desta forma, os componentes femorais demandam mais planejamento devido a influência nos espaços articulares caso seja necessário modificação do dimensionamento AP, principalmente nos homens brasileiros.

Cirurgiões devem se atentar para risco de invasão de corte ( *notching* ) na cortical anterior quando a divergência de medida AP for maior que 3 mm devido ao maior risco de fraturas periprotéticas. 19 Nenhum implante femoral feminino apresentou média maior que 3 mm. No entanto, os resultados de fêmures masculinos demandam atenção, já que 10 implantes (Scorpio, Sartori, Ortosintese, MetaBio, PAR, Baumer, NG, Sigma, Nex Gen, Indus) apresentaram divergência na maioria das amostras ósseas maiores que 3 mm comparados ao melhor implante AP, aumentando risco de invasão do corte cortical a depender do tipo de guia utilizado no procedimento.

Em relação a divergência na acomodação dos implantes femorais para a medida ML, observou-se maior relevância clínica nas mulheres, com valores médios para a cobertura ideal variando em 4,22 mm. Isso indica que os implantes estudados apresentam relação de acomodação ML menos “customizadas” para os fêmures das mulheres brasileiras se comparado aos homens. Os resultados reforçam a necessidade de implantes estreitos para favorecer essa população. A presença de implantes sobre aumento mediolateral é um fato clínico relevante, pois cirurgiões podem precisar subdimensionar o implante a fim de não proporcionar contato com estruturas capsuloligamentares periarticulares que potencializam dor pós-operatória. Ressalta-se que, quando subdimensionar o implante femoral é necessário para acomodação ML influencia diretamente nos dimensionamentos dos espaços articulares, principalmente o de flexão.

A relação ML dos fêmures de homens foram mais largas comparado às mulheres. Esta mesma característica foi demonstrada na população hispânica no estudo de McNamara et al. 12 Diante destes achados, seria oportuno que existissem implantes estreitos que sustentam a mesma distância AP com menor dimensão ML, assim como implantes largos com a mesma distância AP e distâncias ML mais alongadas. Isso ofereceria melhor acomodação óssea a depender da variação de cada paciente, já que a falta de cobertura óssea ML femoral leva a apoios distantes das corticais. Apoio em osso esponjoso com menor resistência mecânica potencializa complicações, como afundamento ósseo tanto no fêmur quanto na tíbia.

Vale notar que as diferenças médias milimétricas para a distância ML ficaram abaixo de 5 mm, no geral. Essa distância distribuída nos compartimentos medial e lateral não representa relevância clínica para desfecho, sendo aceitável como sub- ou sobredimensionamento do implante sem comprometimento técnico no fêmur e tíbia.

Em relação aos resultados observados para os implantes tibiais observou-se que os implantes se acomodam melhor nas anatomias das mulheres comparado aos homens, principalmente devido à distância AP. Ainda que possa diminuir o apoio cortical posterior e, consequentemente, expor o implante a qualidade óssea esponjosa, não houve preocupação com hiperdimensionamento, no qual o implante está em contato com estruturas capsulares e tendíneas posteriores, podendo provocar desconforto e dor pós-operatória. 20 21

Estudos sobre os aspectos morfológicos dos joelhos populacionais são amplamente observados com resultados satisfatórios para o desfecho adaptativo anatômico. Yue et al. 22 Cheng et al. 5 9 estudaram as relações antropométricas tibiais proximais asiáticas e compararam com outras populações, demonstrando diferenças morfológicas articulares. Além disso, Vaidya et al. 23 Urabe et al. 24 e Chaichankul et al. 25 estudaram as relações dos componentes de ATJ com as morfologias epifisárias nas populações indiana, japonesa e tailandesa, demonstrando boa relação de correspondência de mensuração. Desta forma, ainda que existam variações anatômicas observadas nas diferentes populações mundiais para uma customização plena dos implantes disponíveis para a ATJ, os dimensionamentos disponíveis atendem, de maneia geral, a cobertura óssea e funcionalidade.

Ainda assim, Ho et al. 26 encontraram que a relação de alguns implantes pode não ser satisfatória para a população chinesa *.* Em relação ao ranqueamento dimensional AP versus ML tanto para o componente femoral e tibial observou-se destaque para os implantes importados com as melhores proporções para ambos os sexos. Em relação aos implantes nacionais, destacou-se o implante Rotaflex, ranqueado na sétima e sexta posição para mulheres e homens. Neste contexto, observa-se oportunidade de otimização para o desenvolvimento morfológico de implantes nacionais a fim de melhorar o “vestimento” articular para nossa população.

O presente estudo possui limitações e os resultados devem ser analisados com senso crítico. Não foram analisados os perfis biométricos de altura e peso dos pacientes, atrapalhando nossa compreensão de como as divergências anatômicas da população podem estar relacionadas a outras características biotípicas, assim como as variações de tamanho articulares que poderiam influenciar na customização em acordo com os números de componentes de cada fabricante.

Adicionalmente, não foram analisados os perfis de miscigenação das amostras para determinar se os grupos estudados poderiam ter vieses de raça ou cor. Os cortes ósseos parametrizados no estudo radiológico podem não corresponder aos operativos, a depender das condições cirúrgicas (hipoplasias ou defeitos ósseos). Essa divergência agregaria viés de transposição aos resultados.

Outro fator limitante do estudo foi não avaliar as medidas tibiais AP laterais em implantes assimétricos. É possível que os implantes anatômicos podem apresentar melhores acomodações laterais que os implantes não anatômicos, fato não analisado.

As relações de intercambialidade entre os implantes femorais e tibiais para os casos de divergências de dimensionamento ósseo nos pacientes também não foram analisadas.

O ranqueamento realizado corresponde aos achados morfológicos da população analisada e pode não corresponder a toda população brasileira.

## Conclusão

Os implantes estudados apresentam satisfatória cobertura óssea articular dos joelhos de população brasileira, com pequenas variações de dimensões. Entretanto, foram encontrados resultados com diferenças maiores do que 10 mm. Implantes importados se demonstraram mais customizáveis que os brasileiros em nossa população.
